# Clinical effectiveness and safety of amlodipine/losartan‐based single‐pill combination therapy in patients with hypertension: Findings from real‐world, multicenter observational databases

**DOI:** 10.1111/jch.14380

**Published:** 2021-10-29

**Authors:** Jieun Lee, Jaeyun Choi, Yunjin Yum, Hyung Joon Joo, Yong‐Hyun Kim, Hyonggin An, Eung Ju Kim

**Affiliations:** ^1^ Division of Cardiology Department of Internal Medicine Korea University Guro Hospital Seoul Republic of Korea; ^2^ Department of Biostatistics Korea University College of Medicine Seoul Republic of Korea; ^3^ Division of Cardiology Department of Internal Medicine Korea University Anam Hospital Seoul Republic of Korea; ^4^ Division of Cardiology Department of Internal Medicine Korea University Ansan Hospital Ansan Republic of Korea

**Keywords:** amlodipine, chlorthalidone, losartan, rosuvastatin, single‐pill combination

## Abstract

Various single‐pill combinations (SPCs) have been introduced to improve drug compliance and clinical efficacy. However, there is a lack of real‐world evidence regarding the effectiveness of these SPCs for hypertension. This study evaluated the real‐world clinical efficacy and safety of amlodipine/losartan‐based SPC therapies in patients with hypertension in a real‐world setting. A total of 15 538 patients treated with amlodipine/losartan‐based SPCs [amlodipine + losartan (AL), amlodipine + losartan + rosuvastatin (ALR), and amlodipine + losartan + chlorthalidone (ALC)] were selected from the database of three tertiary hospitals in Korea. The efficacy endpoints were target blood pressure (BP) and low‐density lipoprotein cholesterol (LDL‐C) achievement rates. Safety was evaluated based on laboratory parameters. Drug adherence was defined as the proportion of medication days covered (PDC). The target BP attainment rate was above 90% and was similar among the three groups. Although many patients in the AL and ALC groups took statins, the target LDL‐C attainment rate was significantly higher in the ALR group than in the AL and ALC groups. Safety endpoints were not significantly different among the groups, except serum uric acid level and incidence rate of new‐onset hyperuricemia, which were significantly lower in the AL and ALR groups than in the ALC group. The PDC was > 90% in all groups. In the real‐world hypertensive patients, amlodipine/losartan‐based SPC therapy demonstrated good target BP achievement rates. Especially, rosuvastatin‐combination SPC showed better target LDL‐C goal achievement rate compared to the other SPCs. All three amlodipine/losartan‐based SPC had excellent drug adherence.

## 1 INTRODUCTION

The prevalence of hypertension is continuously increasing and is expected to reach approximately 1.56 billion worldwide by 2025.^1^ In Korea, the number of patients with hypertension is continuously increasing and has exceeded 12 million; however, only 9 million take antihypertensive agents and only 6.5 million of them regularly visit a physician for treatment.^2^ The maintenance of appropriate blood pressure (BP) [systolic BP (SBP) < 140 mmHg or diastolic BP (DBP) < 90 mmHg] in patients with hypertension undergoing treatment is a challenging issue to date. Many patients with hypertension require at least two antihypertensive drugs to achieve their target BP.^3^ In Korea, 43.2% of the patients undergoing treatment for hypertension take two antihypertensive drugs, and 16.1% are on three or more antihypertensive medications.^2^ Moreover, most patients with hypertension take additional medications for the comorbidities, among which, dyslipidemia is one of the most common. Thus, 53.8% of the patients undergoing treatment for hypertension also take antilipidemic medication.

Pill burden in these patients may lead to non‐adherence to drug intake, which makes it difficult to control BP as well as overall cardiovascular risk factors. A meta‐analysis revealed that poor drug adherence was associated with an increased risk of stroke in patients with hypertension.^4^ Thus, the 2018 European guidelines for hypertension emphasized that drug adherence is an important factor in BP management and recommended the preemptive use of single‐pill combinations (SPCs) to simplify drug regimens.^5^

Among drug combination regimens, the combination of renin–angiotensin system inhibitors and calcium channel blockers (CCBs) has been studied for their cardiovascular protection.^6^ In addition to cardiovascular protection, angiotensin II receptor blockers (ARBs) have comparable BP‐lowering effects and improve peripheral edema caused by CCBs.^7^ In Korea, 61.1% of the patients with hypertension and taking two antihypertensive medications are treated with a combination of renin–angiotensin system inhibitors (in particular, ARBs) and CCBs.^2^ Amlodipine (68%), among CCBs, and losartan (27%), among ARBs, are the most commonly prescribed antihypertensive agents in each class, according to the Korean healthcare data system. The combination of these two drugs have has been shown to be effective and safe for hypertension management.^8^, ^9^ In addition, amlodipine/losartan‐based FDCs have the second longest prescribed history among CCB/ARB‐based SPCs in Korea, and they also have the highest prescribed rates among the CCB/ARB‐based SPCs in three tertiary hospitals of our study. Moreover, amlodipine/losartan‐based SPCs have strong line‐up combinations by being available as amlodipine/losartan FDCs for general hypertensive patients, amlodipine/losartan/chlorthalidone for patients with resistant hypertension, and amlodipine/losartan/rosuvastatin FDCs for patients with both hypertension and dyslipidemia, which provide great options to evaluate the efficacy and safety of FDCs for hypertensive patients with broad clinical spectrums.^10^, ^11^

Prior studies on amlodipine/losartan‐based SPCs [amlodipine + losartan (AL), amlodipine + losartan + chlorthalidone (ALC), and amlodipine + losartan + rosuvastatin (ALR)] were limited by the small sample size (less than 200 participants) and the relatively short follow‐up periods (less than 8 weeks). Further, there has been no large‐scale observational study on the long‐term clinical benefits of these three types of amlodipine/losartan‐based SPCs. In this study, the clinical efficacy, safety, and drug adherence of the three SPCs were compared in a real‐world setting.

## 2 METHODS

### 2.1 Study design

This was a multicenter, retrospective, observational, and cohort study. This study was performed using the Observational Medical Outcomes Partnership (OMOP) Common Data Model (CDM) database of three tertiary hospitals (Korea University Anam Hospital, Korea University Guro Hospital, and Korea University Ansan Hospital) in Korea. The Observational Health Data Sciences and Informatics collaboration provides the OMOP CDM schema, which is being utilized to standardize the electronic health records (EHRs) of hospitals into the OMOP CDM database (https://github.com/OHDSI/CommonDataModel/). In Korea, ICD‐10 code system is used for the diagnosis, and OMOP‐CDM provides unique concept ID mapped to the ICD‐10 code. Thus, the data were analyzed using OMOP‐CDM concept ID, which is mapped to the ICD‐10 code. Detailed OMOP‐CDM concept IDs were provided in the supplementary materials. The OMOP‐CDM data of the present study was stored in Microsoft's structured query language (SQL) Server, and the data were extracted through direct querying using SQL.

**FIGURE 1:**
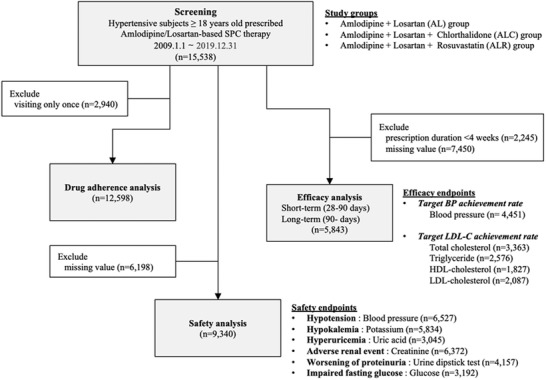
Study schema The safety endpoints were defined as new‐onset hypotension, hypokalemia, hyperuricemia, and impaired renal function based on serum creatinine levels, proteinuria grade, and impaired fasting glucose level. The efficacy endpoints were defined by the target blood pressure and LDL‐C attainment rates. Abbreviations: AL, amlodipine/losartan single‐pill combination group; ALC, amlodipine/losartan/chlorthalidone single‐pill combination group; ALR, amlodipine/losartan/rosuvastatin single‐pill combination group; BP, blood pressure; HDL, high‐density lipoprotein; LDL‐C, low‐density lipoprotein cholesterol; SPC, single‐pill combination

Patients with hypertension were defined as those with SBP ≥ 140 mmHg, DBP ≥ 90 mmHg, or who had been treated with any antihypertensive agents. We screened patients ≥ 18 years who were treated with amlodipine/losartan‐based SPCs at three tertiary hospitals between January 2009 and December 2019 (n = 15 538) (Figure 1. Study schema).

For safety analysis, we included all patients who had been prescribed amlodipine/losartan‐based SPCs at least once (n = 15 538). A total of 13 239 patients were included in the efficacy analysis after excluding patients who had been prescribed amlodipine/losartan‐based combination pills for less than 4 weeks (n = 2245). If a patient was re‐prescribed amlodipine/losartan‐based SPCs after more than one year of “pill‐vacation,” only the re‐prescription data were included.

### 2.2 Efficacy assessment

BP and lipid profiles [total cholesterol, low‐density lipoprotein cholesterol (LDL‐C), high‐density lipoprotein cholesterol (HDL‐C), and triglycerides] were used to assess the efficacy of amlodipine/losartan‐based SPCs. Blood pressure was measured with an automatic sphygmomanometer in a quiet place after resting for at least 5 minutes in a sitting position. Patients were instructed not to smoke, drink alcohol, or consume caffeine within 30 minutes of blood pressure measurement. Short‐term efficacy endpoints were the earliest measurements of BP and lipid profiles between 28 and 90 days after the first prescription. Long‐term efficacy was assessed using the last measurements of BP and lipid profiles at least 90 days after the first prescription. If BP was measured twice on the same day, the average of all measured BP values was used for the analysis. The target BP achievement rate was defined as the proportion of patients who achieved the target BP (SBP < 140 mmHg or DBP < 90 mmHg). Target LDL‐C levels were individually determined based on the cardiovascular risk profile of each patient according to the 2018 Guidelines for the Management of Dyslipidemia in Korea.^12^

### 2.3 Safety assessment

BP and laboratory findings were used to assess the safety of the amlodipine/losartan‐based SPCs. Hypotension was defined as SBP < 90 mmHg. Hypokalemia was defined as serum potassium level ≤ 3.0 mmol/L. Serum uric acid level > 6.5 mg/dL was defined as hyperuricemia. Fasting plasma glucose level > 100 mg/dL was defined as impaired fasting glucose level. Renal adverse event was defined as an increase in serum creatinine level (> 0.4 mg/dL) from baseline. The urine protein dipstick test was used to assess proteinuria, and an individual's urine protein level was defined as “worsening” if the result of urine dipstick test increased by more than one.

### 2.4 Assessment of drug adherence

Adherence to amlodipine/losartan‐based SPCs was assessed using the proportion of days covered (PDC).^13^ PDC was calculated as the total number of medication‐covered days, the sum of the total number of prescription days for drugs during the follow‐up period, divided by the number of days of drug enrollment, which was defined as the period between the first prescription day and the last day of administration of amlodipine/losartan‐based SPCs. Because of the difficulty of tracking the days patients took the prescribed drugs precisely, if the patients revisited to refill their drugs, it was considered that the patients have had taken all of the previously prescribed medications. Pill splitting or doubling of pills from previous prescriptions was not considered. In the case of an early refill, the covered days were calculated by adjusting the number of overlapping days.

### 2.5 Other definitions

Diabetes mellitus was defined when patients had been diagnosed with International Classification of Diseases‐10 (ICD‐10) codes E10–E14, treated with oral hypoglycemic agents or insulin, had fasting plasma glucose level ≥ 126 mg/dL, or had HbA1c ≥ 6.5% at screening. Patients were diagnosed with dyslipidemia according to the ICD‐10 codes E78.0–78.6, E78.8, E78.9, E88.8, and E88.9, or if they were treated with any lipid‐lowering agents. Patients with an estimated glomerular filtration rate (eGFR) < 60 mL/min/1.73 m^2^, as calculated using the Modification of Diet in Renal Disease study formula, were diagnosed with chronic kidney disease. Other comorbidities were defined according to the ICD‐10 codes: coronary artery disease (I20.0, I21, I22, and I25.2), atherosclerotic ischemic stroke/transient ischemic attack (TIA; I63, I64, and G45), peripheral vascular disease (I70.0, I70.1, I70.2, I70.8, and I70.9), carotid artery disease (I65.2), and abdominal aortic aneurysm (I71.3, I71.4, I71.5, I71.6, I71.8, and I71.9). Smoking status and alcohol consumption were defined using the concept IDs in the OBSERVATION table of OMOP‐CDM database. The other OMOP‐CDM concept IDs for laboratory tests or medications were also described in the supplementary materials.

### 2.6 Statistical analyses

Data are expressed as mean ± standard deviation for continuous variables and as n (%) for categorical variables. Chi‐square test and analysis of variance were used to compare categorical and continuous variables, respectively, in the three treatment groups. Post hoc analysis was performed to compare each pair of groups using Bonferroni correction. Changes in BP, lipid profile, and laboratory parameters were compared using paired *t*‐tests. Statistical analyses were conducted using all available data without imputing missing values. For non‐normally distributed variables, Wilcoxon sign‐rank test and Kruskal‐Wallis test were performed instead of paired *t*‐tests and ANOVA, respectively. All tests were two‐sided at a significance level of 0.05. All statistical analyses were performed using Statistical Analysis Software, version 9.4 (SAS Institute, Cary, NC, USA).

## 3 RESULTS

The baseline characteristics of the study participants are presented in Table 1. The mean age of the study population was 63.3 ± 13.7 years, and 56.7% of the study population were males. Baseline characteristics were different among the groups, suggesting that there were preferred patients in different treatment groups. The mean age of the ALR group was higher than that of the other two groups. Poor lifestyle behaviors, including smoking and drinking, were worse in the AL group. Cardiovascular comorbidities (dyslipidemia, coronary artery disease, stroke, or TIA) were more prevalent, in addition to higher body mass index, in the 3‐drug combination pill groups (ALC and ALR groups) than in the AL group. In addition, chronic kidney disease was less frequently observed in the ALR group than in the other two groups. More than 50% of the patients in all groups were taking antihypertensive medications before the start of amlodipine/losartan‐based SPCs; in particular, the proportion of such patients was the highest in the ALC group. In contrast, the proportion of patients who were previously taking antilipidemic medications before starting amlodipine/losartan‐based SPCs was the lowest in the ALR group (rosuvastatin combination group). The total number of pills consumed by patients was significantly lower in the three‐drug combination pill groups (ALC and ALR groups) than in the two‐drug combination pill group (AL group) (*p* < .01).

**TABLE 1:** Baseline demographic characteristics

	Total population	AL group	ALC group	ALR group	
	(no. = 15 538)	(no. = 13 331)	(no. = 955)	(no. = 1252)	*p*
Men, no. (%)	8803 (56.7)	7554 (56.7)	556 (58.2)	693 (55.4)	.40
Age (year)	63.3 ± 13.7	63.2 ± 13.7^b)^	63.2 ± 14.0^c)^	65.4 ± 12.4^b),c)^	<.01
BMI (kg/m^2^)	25.5 ± 4.1	25.3 ± 4.1^a),b)^	27.1 ± 4.8^a),c)^	26.2 ± 3.9^b),c)^	<.01
Smoker, no. (%)	1460 (9.4)	1358 (10.2)^a),b)^	47 (4.9)^a)^	55 (4.4)^b)^	<.01
Alcoholic, no. (%)	1832 (11.8)	1702 (12.8)^a),b)^	63 (6.6)^a)^	67 (5.4)^b)^	<.01
Dyslipidemia, no. (%)	7732 (49.8)	6274 (47.1)^a),b)^	657 (68.8)^a)^	801 (64.0)^b)^	<.01
Diabetes mellitus, no. (%)	5888 (37.9)	5013 (37.6)	376 (39.4)	499 (39.9)	.18
CKD, no. (%)	2224 (34.9)	2016 (35.4)^b)^	96 (34.8)	112 (27.9)^b)^	.01
Proteinuria ≥ 1+, no. (%)	1265 (30.4)	1173 (30.4)	38 (31.7)	54 (29.8)	.94
CAD, no. (%)	1723 (11.1)	1365 (10.2)^a),b)^	184 (19.3)^a),c)^	174 (13.9)^b),c)^	<.01
Stroke or TIA, no. (%)	1398 (9.0)	1075 (8.1)^a),b)^	138 (14.5)^a)^	185 (14.8)^b)^	<.01
Prior antihypertensive medication, no. (%)	8692 (55.9)	7374 (55.3)^a)^	625 (65.4)^a),c)^	693 (55.4)^c)^	<.01
Prior antilipidemic medications, no. (%)	6954 (44.8)	5865 (44.0)^a),b)^	615 (64.4)^a),c)^	474 (37.9)^b),c)^	<.01
Total number of pills	4.2 ± 3.5	4.4 ± 3.7^a),b)^	3.4 ± 2.7^a)^	3.2 ± 2.4^b)^	<0.01

a), b), and c) represent statistically significant difference at α = 0.05 between AL vs ALC, AL vs ALR, and ALC vs ALR, respectively. Values are presented as the mean ± standard deviation or no. (%).

Abbreviations: AL, amlodipine + losartan; ALC, amlodipine + losartan + chlorthalidone; ALR, amlodipine + losartan + rosuvastatin; BMI, body mass index; CKD, chronic kidney disease; CAD, coronary artery disease; TIA, transient ischemic attack.

**FIGURE 2:**
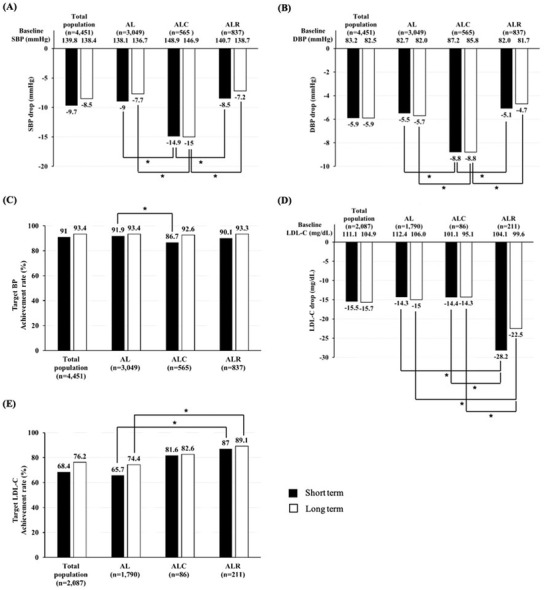
Efficacy of amlodipine/losartan‐based SPCs (A) changes in SBP, (B) changes in DBP, (C) target BP achievement rate, (D) changes in LDL‐C, (E) target LDL‐C achievement rate. * indicates statistically significant difference at α = 0.05. Abbreviations: AL, amlodipine + losartan ; ALC, amlodipine + losartan + chlorthalidone; ALR, amlodipine + losartan + rosuvastatin; BP, blood pressure; SBP, systolic blood pressure; DBP, diastolic blood pressure; LDL‐C, low‐density lipoprotein cholesterol

The mean follow‐up duration was 49.2 days for the short‐term efficacy analysis and 330.0 days for the long‐term analysis. Both SBP and DBP were significantly reduced during the short‐term efficacy analysis (−9.7 mmHg for SBP and −5.9 mm g for DBP, *p* < .05) (Figure 2). Reduction in BP during short‐term analysis was maintained in the long‐term analysis (−8.5 mm Hg for SBP and −5.9 mm Hg for DBP, *p* < .05). Among all groups, the ALC group had the highest baseline SBP and DBP; therefore, despite the greatest decrease in BP in the ALC group, SBP was still higher in the ALC group than in the other two groups during the short‐term efficacy analysis. Notably, during the long‐term efficacy analysis, these differences were reduced and target BP attainment rates were similar among the three groups; this may be due to the remarkable BP‐lowering effect observed in the ALC group. In Conclusion, the target BP attainment rate of amlodipine/losartan‐based SPCs was 93.4% in the long‐term efficacy analysis.

Baseline blood lipid profiles were found to differ among the groups. Total cholesterol, LDL‐C, and triglyceride levels were significantly improved in all groups (*p* < .05). In the long‐term efficacy analysis, total cholesterol level was 23.7 mg/dL lower than the baseline in the ALR; however, in the AL group and ALC group, total cholesterol levels were 16.2  and 17.6 mg/dL, respectively. Similarly, LDL‐C level was 22.5 mg/dL lower than the baseline in the ALR group in the long‐term efficacy analysis, whereas the differences in the AL and ALC groups were 15.0  and 14.3 mg/dL, respectively. The ALR group had the lowest total cholesterol and LDL‐C levels among the three groups during both the short‐ and long‐term follow‐up periods. Moreover, the target LDL‐C attainment rate was the highest in the ALR group during both the short‐term and long‐term analyses. Notably, a significant proportion of patients in the AL and ALC groups were also taking statins (38.9% and 61.2%, respectively). We performed a separate analysis for patients taking statins in the AL and ALC groups and patients in the ALR group. The target LDL‐C attainment rate of the ALR group was the highest (89.1%) during the long‐term efficacy analysis. The AL and ALC groups had rates of 74.8% and 83.6%, respectively (*p* < .01).

Table 2 presents the safety profiles of the three groups. The incidence rate of new‐onset hypotension did not significantly differ among the groups. The baseline serum potassium level was slightly lower during follow‐up in the ALC group than in the other groups. The incidence rate of new‐onset, severe hypokalemia (< 3.0 mmol/L) was statistically insignificant. The follow‐up serum uric acid level and the incidence rate of new‐onset hyperuricemia were significantly lower in the AL and ALR groups than in the ALC group. Although follow‐up plasma glucose levels were higher in the ALC group than in the other groups, the incidence rates of new‐onset impaired fasting glucose were not significantly different among the groups. Baseline and follow‐up serum creatinine levels were higher in the AL group than in the other groups. The incidence rate of a clinically significant increase in serum creatinine level and worsening of proteinuria grade were statistically insignificant among the groups.

**TABLE 2:** Safety profiles of amlodipine/losartan‐based SPCs

		Total population	AL group	ALC group	ALR group	*p*
New‐onset hypotension, no. (%)		8 (0.1)	4 (0.1)	2 (0.3)	2 (0.2)	.28
Potassium	Baseline (mmol/L)	4.3 ± 0.5	4.2 ± 0.5^b)^	4.3 ± 0.5^c)^	4.4 ± 0.5^b),c)^	<.01
	Follow‐up (mmol/L)	4.2 ± 0.5	4.2 ± 0.5^a),b)^	4.1 ± 0.5^a),c)^	4.4 ± 0.5^b),c)^	<.01
	New‐onset hypokalemia, no. (%)	30 (0.5)	26 (0.5)	3 (1.2)	1 (0.3)	.22
Uric acid	Baseline (mg/dL)	5.6 ± 1.9	5.6 ± 1.9	5.5 ± 1.7	5.6 ± 1.8	.78
	Follow‐up (mg/dL)	5.3 ± 1.8	5.3 ± 1.8^a),b)^	5.7 ± 1.9^a),c)^	4.9 ± 1.5^b),c)^	<.01
	New‐onset hyperuricemia, no. (%)	211(6.9)	186 (6.9)^a)^	17 (13.0)^a),c)^	8 (3.9)^c)^	<.01
Glucose	Baseline (mg/dL)	127.1 ± 50.3	127.1 ± 50.6	130.3 ± 45.0	124.6 ± 50.9	.56
	Follow‐up (mg/dL)	125.3 ± 49.2	124.5 ± 46.8^a)^	144.3 ± 87.9^a),c)^	121.6 ± 38.5^c)^	<.01
	New‐onset IFG, no. (%)	408 (11.3)	371 (11.7)	20 (11.4)	17 (7.2)	.11
Creatinine	Baseline (mg/dL)	1.26 ± 1.4	1.29 ± 1.5^a),b)^	0.97 ± 0.4^a)^	1.07 ± 0.9^b)^	<.01
	Follow‐up (mg/dL)	1.27 ± 1.5	1.29 ± 1.5^a),b)^	1.07 ± 0.6^a)^	1.10 ± 1.0^b)^	<.01
	Increase ≥ 0.4 mg/dL, no. (%)	304 (4.8)	273 (4.8)	15 (5.4)	16 (4.0)	.67
Worsening of proteinuria grade, no. (%)		530 (12.8)	485 (12.6)	12 (9.92)	33 (18.1)	.06

Values are presented as the mean ± standard deviation or no. (%). a), b), and c) indicate statistically significant differences at α = 0.05 between AL vs ALC, AL vs ALR, and ALC vs ALR, respectively.

Abbreviations: AL, amlodipine + losartan; ALC, amlodipine + losartan + chlorthalidone; ALR, amlodipine + losartan + rosuvastatin; IFG, impaired fasting glucose; SPC, single‐pill combination.

**FIGURE 3:**
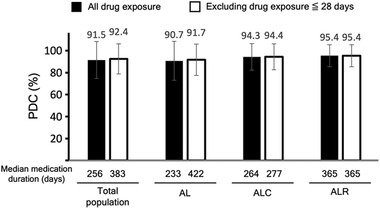
Proportion of days covered for amlodipine/losartan‐based SPCs Abbreviations: AL, amlodipine + losartan; ALC, amlodipine + losartan + chlorthalidone; ALR, amlodipine + losartan + rosuvastatin; PDC, proportion of days covered; SPC, single‐pill combination

The PDC for the amlodipine/losartan‐based SPCs is shown in Figure 3. The PDC was ≥ 90% in all groups. The median duration of drug exposure was 233–422 days. Notably, PDC in the two‐drug combination group (AL group) was significantly lower than that in the three‐drug combination groups (ALC and ALR groups; *p* < .05). Furthermore, the proportion of patients with PDC > 80% was lower in the 2‐drug combination group (AL group) than in the 3‐drug combination groups (ALC and ALR groups; 85.6%, 90.5%, and 93.2%, respectively; *p* < .05).

## 4 DISCUSSION

This study was carried out to determine the efficacy and safety profiles of amlodipine/losartan‐based SPCs in patients with hypertension in a real‐world clinical setting. This study is the first and the largest real‐world long‐term observational study of the three amlodipine/losartan based SPCs using electronic health record data. From this study, we found several clinical perspectives for hypertensive patients. First, the three‐drug combination pill groups (ALC and ALR groups) had a lower total pill burden and higher drug adherence than the two‐drug combination pill group (AL group). Total pill number was lowered more than 1 in the three‐drug combination pill groups compared to the two‐drug combination pill group. The PDC in the three‐drug combination groups was higher compared to the two‐drug combination group (95.0% vs 90.7%). Second, all three groups treated with amlodipine/losartan‐based SPCs had similar excellent BP control despite different baseline characteristics. Target BP achievement rates were more than 90% in all three groups. Third, ALR group of statin‐containing SPC displayed the greatest benefits for dyslipidemia control. Target LDL‐C achievement rate was the highest as 89.1% in the ALR group. Forth, all three groups showed good safety profiles.

The target BP attainment rate of amlodipine/losartan‐based SPCs during long‐term assessment was approximately 90%, which is considerably higher than the average BP control rate of patients with hypertension and undergoing treatment in Korea (71%).^14^ Surprisingly, in developed countries, such as Canada, Germany, and the United States of America, only 70% to 85% of patients undergoing treatment for hypertension have controlled BP.^15^ These findings suggest an increase in medication compliance due to reduced pill burden in amlodipine/losartan‐based SPC therapy for patients with hypertension who were already receiving multidrug therapy. Previous studies have shown that fewer pills are associated with better drug adherence and hypertension control.^16^, ^17^ Moreover, a recent systematic review including 44 studies concluded that SPC improves drug adherence and contributes to superior BP control in patients with hypertension.^18^ The PDC for amlodipine/losartan‐based SPCs in this study was 91.5%, which was either comparable to or higher than that reported in previous studies on SPCs.^19^, ^20^, ^21^

Because dyslipidemia is a chronic disease associated with no symptoms and has a slow progression, many patients with dyslipidemia do not fully understand the need for treatment. As a result, adherence to treatment is low. In addition, as patients with hypertension are already taking several medications, there is a reluctance to take additional medications to treat dyslipidemia. Indeed, prior studies have reported that 35–75% of treated patients with hypertension and dyslipidemia were not prescribed any antilipidemic medication.^22^, ^23^ As lowering the pill burden motivates drug adherence, statin‐containing SPCs could be an attractive treatment option to improve lipid control in patients taking multiple pills. In this study, 38.9% of the patients with hypertension treated with amlodipine/losartan SPC (AL group) were prescribed additional pills for dyslipidemia. Notably, the target LDL‐C attainment rate in these patients was lower than that in patients treated with amlodipine/losartan/rosuvastatin (ALR group: 74.8% vs 89.1%). In addition, 62.1% of the patients in the ALR group started taking statins in amlodipine/losartan/rosuvastatin SPC, suggesting that statin‐containing SPCs could promote treatment and improve the dyslipidemia control rate in patients with hypertension.

Inflammation plays an important role in the pathophysiology of hypertension. Like C‐reactive protein (CRP), uric acid has been suggested as one of the surrogate markers for inflammation in hypertensive patients. Serum uric acid level is independently associated with CRP in the patient with hypertension.^24^, ^25^ Serum uric acid level is usually increased during follow‐up in hypertensive patients, and hyperuricemia is known to adversely affect hypertension as well as the other cardiovascular diseases. Losartan decreases serum uric acid levels through uricosuric action via the URAT1 inhibition.^26^ Thus, compared with the other ARB‐based SPCs, losartan‐based SPCs showed beneficial effects in the patients with hypertension. The present study also demonstrated the serum uric acid‐lowering effect of losartan‐based SPCs except the ALC group. Diuretics, including chlorthalidone, are known to increase serum uric acid levels. Although the incidence rate of new‐onset hyperuricemia was higher in the ALC group than in the other groups (13% vs 6.9% vs 3.9%, hyperuricemia cut‐off level: 6.5 mg/dL), the increase in serum uric acid level in the ALC group was statistically insignificant (0.2 mg/dL, *p* = .12). Previous studies revealed an increase in serum uric acid levels (mean change: 1.28–1.39 mg/dL) and the incidence rate of hyperuricemia (12.5–31.2%, hyperuricemia cut‐off level: 7.2–10.5 mg/dL) with azilsartan/chlorthalidone SPCs.^27^, ^28^ Based on these findings, the results of this study are crucial with respect to the uric acid‐lowering effect of losartan in combination with chlorthalidone. In addition, although the present study could not analyze symptomatic adverse events, amlodipine/losartan SPCs in previous clinical trials showed only mild adverse symptoms (mainly dizziness, headache, and gastrointestinal symptoms) with relatively low (7%) incidence rates.^11^

Our study has several limitations. First, many patients were excluded due to the missing values. Patients analyzed in this study might have higher cardiovascular risks considering that elderly patients or patients with cardiovascular comorbidities might have little resistance to medical tests. Second, the baseline characteristics of the three groups were significantly different, which might be due to the physician's and patient's preferences or different prescription indications. Physicians prescribed ALC to the patients with higher blood pressure and ALR to the patients with higher cardiovascular risks. Therefore, a selection bias may have been present. Multivariate logistic regression analysis showed that ALR would be an independent predictor for the target LDL‐C achievement after adjusting the baseline characteristics (odd ratio 2.32, 95% confidence interval 1.09–4.93, *p* = .03). However, it is more worthwhile to elaborate the clinical efficacy, safety, and drug adherence of the three amlodipine/losartan‐based SPCs than to compare among these three agents. As there are various clinical spectrums of hypertensive patients, the real‐world clinical data of the three different SPCs of the present study will be able to precisely identify the strengths and weaknesses of each SPC and further improve the quality of clinical treatment for the patients with hypertension.

## 5 CONCLUSIONS

To the best of our knowledge, this is the first real‐world long‐term observational study of three amlodipine/losartan‐based SPCs. Amlodipine/losartan‐based SPC therapy demonstrated good target BP achievement rates. Rosuvastatin‐combination SPC showed better target LDL‐C goal achievement rate compared to the other SPCs. All three amlodipine/losartan‐based SPCs showed good efficacy, safety, and excellent drug adherence. Altogether, the findings of the present study will provide guidance for reframing the detailed clinical applications of SPC in patients with hypertension taking multiple pills.

## CONFLICT OF INTEREST

All authors declare no conflict of interests.

## AUTHOR CONTRIBUTIONS

Concept and design: Eung Ju Kim, Hyung Joon Joo.

Acquisition, analysis of data: Hyung Joon Joo, Jaeyun Choi, Yunjin Yum.

Drafting of the manuscript: Hyung Joon Joo, Jaeyun Choi, Jieun Lee.

Critical revision of the manuscript for important intellectual content: Eung Ju Kim, Hyonggjin An, Hyung Joon Joo, Yong‐Hyun Kim.

Obtained funding and administrative, technical, or material support: Eung Ju Kim, Hyung Joon Joo, Yong‐Hyun Kim.

Supervision: Eung Ju Kim, Hyung Joon Joo.

All authors interpreted the data used in this study and reviewed the final version of the manuscript.

## Supporting information

Supporting Information
