# Supplementary material for: Clinical effectiveness and safety of amlodipine/losartan‐based single‐pill combination therapy in patients with hypertension: Findings from real‐world, multicenter observational databases
Source: J Clin Hypertens (Greenwich). 2021 Oct 29;23(11):1975–83. doi: 10.1111/jch.14380 (PMC8630602; doi:10.1111/jch.14380)
Supplement: Supplementary file 1 — Supporting Information [file JCH-23-1975-s001.docx]

**Supplemental data**

**Concept set definitions**

| Concept set | OMOP CDM concept_id |
| --- | --- |
| AL | 42950421, 42950354, 42950393, |
| ALC | 42950866, 42950863, 42950869 |
| ALR | 42969118, 42969112, 42969115, 42969109, 42969102, 42969106 |
| Antidiabetic drug | 19021312, 1529352, 40164943, 43013911, 43013924, 42953740, 42953698, 42953917, 42953818, 19122366, 19122367, 19122368, 1545997, 40164929, 40164939, 19123592, 40164925, 40164885, 40164916, 40164919, 1594976, 42960765, 42960762, 42960759, 44785831, 45775456, 45775620, 42961331, 45774754, 45774893, 46287680, 46287689, 46287408, 46287686, 21130197, 21091002, 19077682, 19059797, 19101729, 21133671, 42960642, 42960645, 42960648, 42961500, 40165997, 40166002, 42942992, 1597772, 1597761, 1597758, 1597773, 19006931, 21169719, 43267262, 21081251, 42962855, 40164891, 40164922, 36887702, 42708168, 42708172, 42708176, 40231394, 40231402, 42960773, 40239218, 42708086, 42708088, 42708090, 40164897, 19106521, 40164946, 40164894, 42962884, 1502829, 19107111, 19107110, 42960653, 43013915, 43013918, 43013896, 43013899, 1525221, 19079293, 1547508, 19079465, 19023425, 19023424, 19023426, 19125041, 19125049, 42961319, 42961322, 42961325, 40166037, 40166041, 42960599, 42960587, 42960590, 42960593, 42961179, 42961189, 42961170, 42961173, 19129179, 19112791, 42902468, 42921644, 42922767, 42922959, 42921783, 42922264, 46234234, 19078603, 19058398, 46233974, 42902587, 42902945, 46233971, 19078552, 46234239, 19078555, 19135264, 43275300, 42902821, 40169222, 19078559, 42902742, 41349138, 41349636, 43518492, 41349148, 41348682, 41348912, 1596972, 46234050, 42902356, 46234047, 46234237, 43297029, 19078558, 42921712, 42921721, 46221558, 35602725, 42961487, 42961484, 42961490, 42961494, 42969162, 42969165, 42969168 |
| Antihypertensive drug | 974447, 974473, 974474, 974642, 974702, 1305450, 1308851, 1308874, 1309071, 1318859, 1318860, 1319942, 1319943, 1321637, 1326020, 1328585, 1328689, 1332419, 1332494, 1332495, 1332497, 1332499, 1332525, 1332527, 1334461, 1334492, 1337070, 1337103, 1340161, 1351559, 1351583, 1351587, 1353780, 1353818, 1353820, 1592858, 19011548, 19011549, 19015804, 19017656, 19019309, 19020063, 19022241, 19022242, 19022948, 19022949, 19023453, 19023454, 19028935, 19028936, 19050220, 19058101, 19073093, 19073094, 19074672, 19074673, 19076924, 19078080, 19078101, 19080128, 19081025, 19096677, 19096678, 19096740, 19096752, 19101748, 19101750, 19101751, 19101807, 19102170, 19102171, 19102491, 19106542, 19106543, 19106593, 19106594, 19107180, 19112606, 19112979, 19112981, 19113063, 19121182, 19122209, 19127432, 19127433, 19127434, 19133612, 19133613, 19134566, 21030573, 21041304, 21056210, 21058150, 21070867, 21097731, 21141332, 21601761, 35604949, 35604953, 35604961, 36883910, 40069686, 40163142, 40163271, 40163275, 40163753, 40163760, 2064553, 2064498, 2064586, 40165762, 40165789, 40167202, 40167843, 40167849, 40167852, 40171661, 40171863, 40171884, 40171905, 40171917, 40174776, 40174811, 40184184, 40184187, 40184217, 40185276, 40185304, 40224166, 40224172, 40224175, 40235487, 40235491, 42707639, 42707641, 42801011, 42801015, 42925744, 42925749, 42925752, 42929931, 42929938, 42929951, 42930392, 42930395, 42932538, 42932541, 42932544, 42932547, 42938510, 42938513, 42938516, 42948648, 42948651, 42948654, 42950300, 42952826, 42955431, 42955514, 42959698, 42959787, 42959791, 42959999, 42960002, 42960005, 42960008, 42960013, 42960018, 42960027,  42960031, 42960035, 42960725, 42960735, 42960852, 42960860, 42960864, 42960868, 42961740, 42961744, 42963311, 42968981, 42968990, 42968999, 42969008, 42969022, 42969031, 42969040, 42969049, 42969058, 42969067, 42969082, 42969085, 42969088, 42969091, 42969094, 42969097, 42969123, 42969126, 42969129, 42969132, 42969135, 42969138, 42969141, 42969149, 42969152, 42969154, 42969157, 42972631, 42972634, 42972637, 42972640, 43275607, 43291777, 44507582, 46287343 |
| Antilipidemic drug | 19029645, 43527029, 43527032, 1545997, 19123592, 1545996, 1545959, 1539469, 1526479, 19022958, 19022174, 44506638, 44506641, 19095325, 1586255, 42969173, 42969232, 42969291, 1526476, 1551865, 19101756, 1551838, 19077244, 42938209, 19077499, 19077498, 19080792, 19019115, 19019116, 19077245, 1551927, 19078988, 1517961, 19129506, 1517991, 19121242, 19051464, 40165642, 40165638, 40165646, 41048773, 40175390, 40175394, 40175400, 19022104, 42968976, 40165245, 40165253, 40165261, 1539411, 1539407, 41085202, 19112569, 46287495 |
| Diabetic melitus | 443732, 4099216, 443731, 443729, 37016354, 43530689, 37017432, 45769905, 45769906, 4193704, 4226121, 45757499, 43530656, 201530, 43530690, 45757277, 43530685, 36717156, 37016768, 4222415, 37016349, 443734, 4228443, 45757363, 37018728, 4063043, 4198296, 4129519, 4196141, 43531010, 4140466, 443412, 4224709, 4225055, 435216, 37017431, 36715571, 4295011, 45769904, 201254, 318712, 200687, 4222553, 377821, 42538169, 4224254, 45763583, 201531, 37016767, 45763584, 45757507, 37018566, 4227210, 37017429, 40484648, 45769876, 4151281, 4063042, 45769832, 4096042, 4221933, 4224879, 4096671, 4096670, 4224419, 4099652, 443733, 4096041,201826 |
| Dyslipidemia | 437530, 437827, 440360, 438720, 437521, 432867, 435516, 437530, 4047784 |
| Coronary artery disease | 321318, 312327, 4108217, 314666 |
| Stroke or TIA | 443454, 381316, 373503 |
| Smoking | 3004518, 4141787, 4041306, 4298794 |
| Drinking | 40481011, 40771103, 4052351, 4074035 |
| Weight | 3025315 |
| Height | 3036277 |
| Systolic BP | 4152194 |
| Diastolic BP | 4154790 |
| Triglyceride | 3022192 |
| Total cholesterol | 3027114 |
| HDL-C | 3007070 |
| LDL-C | 3028437 |
| Potassium | 3023103 |
| Uric acid | 3037556 |
| Creatinine | 3016723 |
| Glucose | 3004501 |
| HbA1c | 40758583 |
| Proteinuria | 3037185 |
